# Ovarian Disease

**Published:** 1845-01

**Authors:** 


*Ovarian Disease.*—Dr. Jeaffreson thus sums up the results of the operation for the extirpation of ovarian tumors in 74 cases:—In 37 cases the tumor was removed and the patient recovered; in 24 the operation was followed by the death of the patient; of these 24 fatal cases, the tumor was removed in 14, could not be removed on account of adhesions in 6, and was found to be other than ovarian tumors in 4 cases. Thus, again, in 74 cases, in which the operation for extirpation of ovarian tumor had been undertaken, it has been completed in 51 instances, in 14 of which it has been followed by death, and in 37 by the successful removal of the tumors, and by recovery of the patients; whilst, out of the 74 cases selected, it was found impossible to carry out the intentions of the operator in 23; or, in other words, the diagnosis was not sufficiently accurate to enable the surgeon to foresee the impracticability of carrying out his intentions. Of these 23 cases, 13 recovered with life, to remain in *statu quo;* 10 died. The cause of failure was, impossibility of removing the tumor, on account of adhesions, in 14 cases; no tumor was found in 3 cases; and the tumor proved to be other than ovarian in 6 instances.—*Boston Med. and Surg. Jour.*, from *London Medical Times.*

